# *Bordetella trematum* bacteraemia secondary to an empyema in an immunocompromised host: A case report and review of the literature

**DOI:** 10.1099/acmi.0.000602.v3

**Published:** 2023-07-13

**Authors:** Crystal Wong, Lynna Grace Calungsud, My-Van La

**Affiliations:** ^1^​ Microbiology, Department of Laboratory Medicine, Changi General Hospital, Singapore, Singapore; ^2^​ Department of Infectious Diseases, Changi General Hospital, Singapore, Singapore

**Keywords:** *Bordetella *infection, bacteraemia, opportunistic infection, empyema

## Abstract

**Introduction.:**

*
Bordetella trematum* infection remains uncommon. More cases of bacteraemia are reported in recent years with the primary infection largely originating from skin and soft tissue sites. Yet, our understanding of its virulence, antibiotic susceptibility profile and treatment is still limited.

**Case presentation.:**

**Case presentation.** We report the first case of *B. trematum* bacteraemia from a left-sided empyema. An 87-year-old female patient with a past medical history of ischaemic heart disease, diabetes mellitus complicated by nephropathy and locally advanced left breast adenocarcinoma presented with fever, productive cough and shortness of breath. The *B. trematum* isolates from blood and pleural fluid were identified by MALDI-TOF and 16S rRNA sequencing. Ceftriaxone and azithromycin commenced empirically on admission were switched to piperacillin-tazobactam after 2 days due to lack of clinical improvement. Despite a pleurocentesis and 1 week of piperacillin-tazobactam with microbiological clearance in blood, the patient continued to deteriorate. Decision to withdraw treatment was made in view of the patient’s prognosis, and the patient succumbed on the fourteenth day of admission. The isolate was susceptible to piperacillin-tazobactam, imipenem and meropenem but had reduced susceptibility or was non-susceptible to cefuroxime, cefotaxime, ceftazidime, cefepime, the aminoglycosides and fluoroquinolones.

**Conclusion.:**

Invasive *B. trematum* infection is associated with significant mortality. Consensus for antibiotic treatment remains unclear, with limited susceptibility data to support specific antibiotic use. We expect more clinical cases will surface with improved microbial identification systems, as well as enhanced clinical awareness. Standardized and more robust susceptibility work are needed to provide clear recommendations and establish consensus in treating invasive infections.

## Data Summary

The *B. trematum* 16S rRNA gene nucleotide sequence, accession number OQ683905 was submitted to GenBank.

## Background

*Bordetella trematum* is an aerobic non-lactose fermenting Gram-negative bacillus, first recognized and described in 1996 by Vandamme *et al*. [1], Most published *B. trematum* cases [2–6] between 2004 to 2019 were based on the recovery of isolates from infected lower limb wounds in the presence of other pathogens, with only a handful of cases recovered from sterile sites. In 2020, Kukla *et al*. [7] described the recovery of *B. trematum* from the respiratory tract of an immunocompromised host, and more recently a letter-to-the-editor by Lee *et al*. [8] described a case of *B. trematum* catheter related bloodstream infection. To date, documented clinical cases are relatively scarce, limited to case series and reports. There are still gaps in our understanding of its pathogenesis and its potential virulence in causing infection.

We present a case of *B. trematum* bloodstream infection secondary to an empyema in an immunocompromised host, and the microorganism’s antibiotic susceptibility profile. To our knowledge, this is the first published case of *B. trematum* bacteraemia from an empyema.

## Case presentation

An 87-year-old female with a past medical history of ischaemic heart disease, diabetes mellitus complicated by nephropathy, and locally advanced left breast adenocarcinoma presented with fever, productive cough and shortness of breath of 5 days' duration. The patient had also complained of reduced urine output during the same period.

At presentation, the patient was afebrile and alert, but tachypnoeic with a respiratory rate of 32 breaths per minute and oxygen saturation was 90 % on room air. Respiratory examination revealed reduced air entry on the left base. The patient’s breast tumour was visible on the left chest, with a discharging blood-stained wound.

Investigations revealed a leucocytosis (92 % neutrophil predominant) of 16.4×10^3^/µl [normal range 4–10×10^3^/µl], haemoglobin of 7.3 g/dl [normal range 13–17 g/dl] and platelet count of 267×10^3^/µl [normal range 150–450×10^3^/µl]. Inflammatory markers were elevated with C-reactive protein (CRP) of 182 mg/l [normal range < 3.0 mg/l] and procalcitonin of 2.54 µg/l [normal range ≤0.5 µg/l]. The patient’s renal function worsened (eGFR of 23 ml min^−1^/1.73 m^2^ from a baseline of 41 ml min^−1^/1.73 m^2^), with urea of 12.4 mmol/l [normal range 2.8–7.7 mmol/l] and creatinine of 167 µmol/l [normal range 65–125 µmol/l]. Sodium level was low at 118 mmol/l [normal range 135–145 mmol/l], with a normal potassium of 4.6 mmol/l [normal range 3.5–5.3 mmol/l]. The liver function test was unremarkable. Admission chest x-ray showed a moderate sized left pleural effusion. In the context of the COVID-19 pandemic and upper respiratory tract symptoms, a nose and throat swab was sent for respiratory pathogens detection. The multiplex PCR (Biofire Respiratory Panel 2.1 Plus) did not detect any viruses including SARS-CoV-2, nor any atypical pathogens (* Bordetella pertussis *, *Bordetella parapertusis*, *Chlamydia pneumoniae* and *Mycoplasma pneumoniae *).

The patient was empirically commenced on intravenous ceftriaxone 2 g once daily and oral azithromycin 500 mg once daily for treatment of complicated pneumonia with type II respiratory failure. With little clinical improvement, on the third day the empiric antibiotics were escalated to intravenous piperacillin-tazobactam 4.5 g twice daily (adjusted according to the patient’s renal impairment).

The aerobic blood culture collected on admission (day 1) flagged positive at 24 hours of incubation in the automated blood culture system, BD BacTec FX System, and Gram stain revealed a Gram-negative bacillus. The following day, culture plates (blood and MacConkey agar) grew oxidase negative, non-lactose fermenting Gram-negative bacilli. Identification was performed by MALDI-TOF Vitek-MS and yielded the organism, *B. trematum *.

On the third day, a left pleurocentesis was performed for diagnostic and symptomatic relief. The appearance of the aspirated pleural fluid was yellow and turbid on macroscopic examination, with a pH of 7.46, cell count of greater than 10 000 cells/mm^3^ (90 % neutrophil predominant), total protein of 26.3 g/l, glucose of 1.0 mmol/l, lactate dehydrogenase (LDH) of 1938 U/l and adenosine deaminase (ADA) of 35.9 U/l [normal range ≤ 30 U/l]. Gram-negative bacilli were seen on direct microscopy, and the pleural fluid cultured pure growth of *B. trematum* the following day, identified by MALDI-TOF. The biochemical, microbiological and clinical findings were in keeping with a left-sided empyema. The microbial identity of both isolates were confirmed by 16S rRNA gene sequencing.

Susceptibility testing was performed using the commercial broth microdilution panel, MicroScan Negative Panel (NM44) and supplemented with antibiotic gradient strip, E-test. The categorical susceptibilities reported were based on the non-Enterobacterales Clinical and Laboratory Standards Institute (CLSI) breakpoints. For the blood isolate, piperacillin-tazobactam was reported as susceptible with a MIC of 4 µg/ml, whilst the cephalosporins (cefotaxime, ceftazidime, cefepime) exhibited high MICs, between 8 to 32 µg/ml, falling within the susceptible and non-susceptible categories. The aminoglycosides (gentamicin, amikacin and tobramycin) and the fluoroquinolones (ciprofloxacin, levofloxacin and moxifloxacin) demonstrated reduced susceptibilities. Tetracycline (MIC <= 4 µg/ml) and trimethoprim-sulfamethoxazole (MIC <= 2 µg/ml) both tested susceptible. Co-amoxiclav and azithromycin were also tested given the clinical experience of these antibiotics used in treating other *Bordetella* species infection, both antibiotics recorded a MIC of 4 µg/ml. (Table 1).

**Table 1.** Case isolate susceptibility results from broth microdilution (MicroScan)

Antibiotic tested	Pleural fluid isolate		Blood culture isolate	
	Susceptibility	MIC (µg/ml)	Susceptibility	MIC (µg/ml)
Ampicillin	–	4	–	8
Ampicillin-sulbactam	–	<=8	–	<=8
Amoxicillin-clavulanic acid	–	<=8	–	4*
Piperacillin-tazobactam	S	<=8	S	4*
Cefuroxime	–	>16	–	>16
Cefotaxime	I	16	R	32
Ceftazidime	S	4	S	8
Cefepime	S	8	I	16
Cefoxitin	R	>16	R	>16
Aztreonam	R	>16	R	>16
Ertapenem	–	<=0.5	–	<=0.5
Imipenem	S	<=2	S	<=2
Meropenem	S	<=1	S	<=1
Ciprofloxacin	I	2	I	2
Levofloxacin	S	<=1	I	4
Moxifloxacin	–	2	–	>2
Amikacin	S	<=8	S	16
Gentamicin	S	4	I	8
Tobramycin	S	4	I	8
Trimethoprim-sulfamethoxazole	S	<=2	S	<=2
Tetracycline	S	<=4	S	<=4
Azithromycin	NP	NP	–	4*

Categorical susceptibilities are interpreted based on other non-Enterobacterales CLSI 2021 breakpoints.

NP not performed.

– No CLSI breakpoints.

*MIC value derived from gradient antibiotic strip (E-test) testing.

**Fig. 1.**
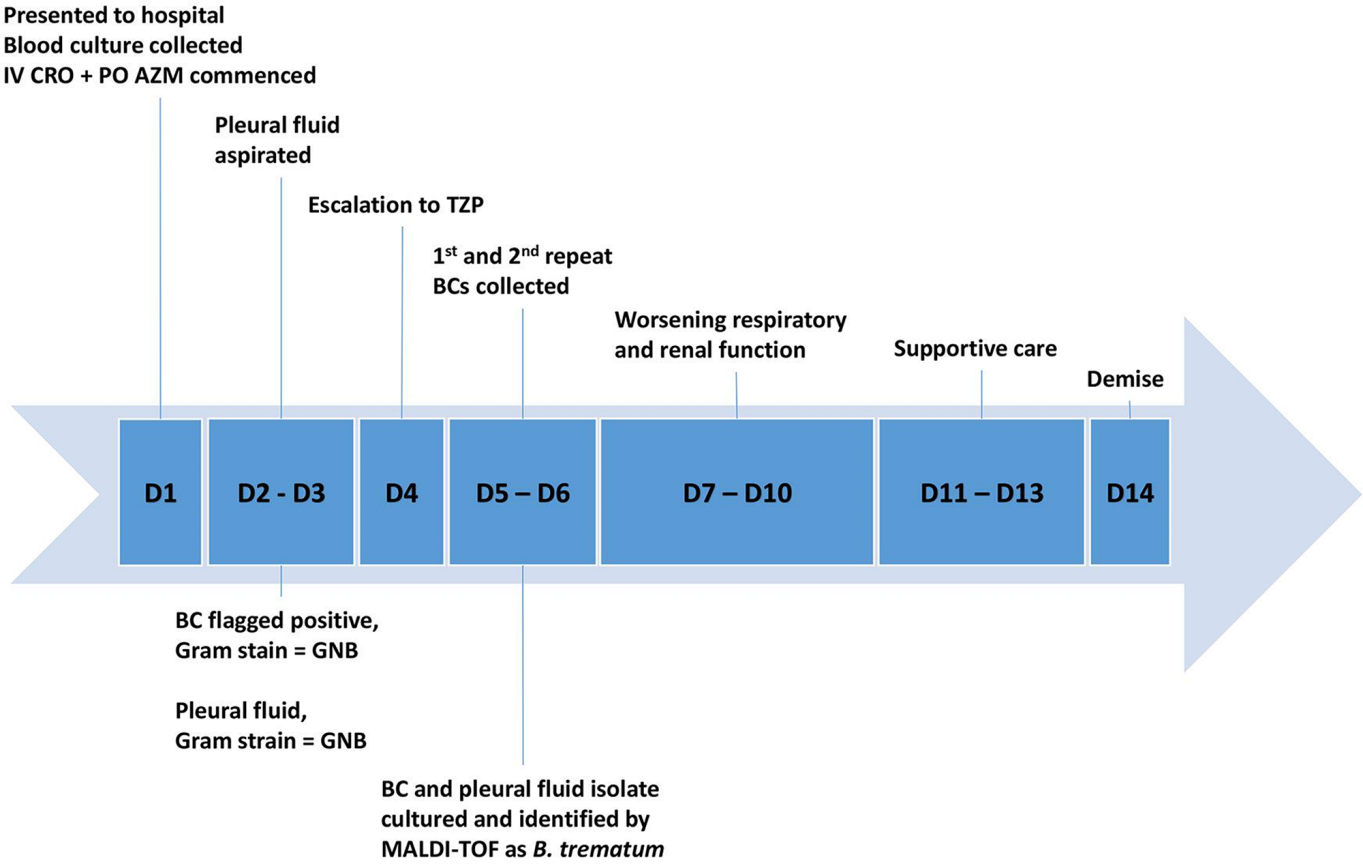
Case chronology. BC, blood culture; GNB, Gram-negative bacilli; IV, intravenous; PO, per oral; AZM, azithromycin; CRO, ceftriaxone; TZP, piperacillin-tazobactam.

Despite active treatment with piperacillin-tazobactam and microbiological clearance in two sets of follow-up blood cultures (collected on day 4 and day 5), the patient’s clinical prognosis maintained guarded with progressively worsening renal function and type II respiratory failure. A collective medical and family decision was made to minimize further active intervention. Supportive and palliative care was instituted and the patient succumbed on the fourteenth day of admission. See Fig. 1 for a summary of events.

## Discussion

Since the discovery and recognition of *B. trematum* from clinical specimens, its pathogenic role in human infection has been debated over the years. Earlier case reports in the literature, were largely from skin and soft tissue infections of the ear, lower limb ulcers and wounds of diabetic patients, which were polymicrobial in nature [2–6]. Some may argue that *B. trematum* recovered from chronic lower limb ulcers or wounds were transient benign colonizers and may not have warranted immediate clinical attention [2]. Furthermore, reports of bloodstream infections [9, 10] and peritonitis [11] were polymicrobial in nature where *B. trematum* was recovered as part of a mixed culture in the sample. In addition, Kulka *et al*. [7] suggested that the recovery of *B. trematum* in the presence of oropharyngeal flora from the respiratory tract (sputum and bronchoalveolar lavage) was a plausible source of infection in an oncology patient.

Two cases of bacteraemia reported by Majewski *et al*. [12] and Lacasse *et al*. [13] had originated from infected skin and soft tissue sites, but resulted in very different clinical outcomes. Majewski *et al*. described a fatal case from a rapidly progressive deep tissue infection of the leg whilst Lacasse *et al*. presented a case with favourable clinical outcome from an upper limb erysipelas in an immunocompromised individual, which resolved completely with antibiotic treatment.

Since 2015, there has been a cumulative number of *B. trematum* bacteraemia cases reported (Table 2). Yet, relatively little is known about the pathogenesis, spectrum of disease and its antibiotic susceptibility profile. Studies have looked at its genomic sequences, described genes encoding the cytolethal distending toxin (CDT) and analysed the O-chain subunit of the lipopolysaccharide (LPS, endotoxin) [14–16]. Novikov *et al*. [17] reported a modification in lipid A of *B. trematum,* which may play a role in evading the host’s immune system.

**Table 2.** Summary of *B. trematum* bacteraemia case reports

	Author and year of report	Age/ sex	Diagnosis, primary infection site	Blood culture	Co-morbidity*	Concomitant pathogen(s) or infection(s)	Treatment†		Clinical outcome
							Empiric	Targeted	
**1**	Halim et al. 2014	60y/M	Septic shock, burns (60 % body)	Mixed with *Enterobacter cloacae *	None	Colonised burns wound with *Klebsiella pneumoniae *, *Pseudomonas aeruginosa* and *Enterobacter sp*.	IMI, NET, CL	IMI, NET, CL	Death
**2**	Saksena *et al*. 2015 [21]	7 m/F	Bacteraemia	Pure	Developmental delay	–	CRO (5d)	TZP/AMI (7d) -> CIP/AZM (5d)	Survived
**3**	Majewski et al. 2016	61y/ Trans-M‡	Septic shock, rapid progressive lower limb wound infection	Pure	Bilateral BKA, DM, CKD, CHD	–	TZP/VAN	TZP/VAN + CIP/CLI + TOB	Death
**4**	Desurmont et al. 2018	65y/F	Bacteraemia, bleeding chest wall (malignant) lesion	Mixed with *Alcaligenes faecalis *	Metastatic breast cancer	–	TZP	TZP (21d)	Survived
**5**	Hummel et al. 2020	75y/F	Septic shock, bilateral lower limb cellulitis	Pure	CHF, HTN, DM	Concurrent *E. coli* UTI (susceptible to antibiotics used).	VAN (1d) + MEM (6d)	AZM/CZ (1d) -> AZM/SXT (1d) -> antibiotic withdrawal	Death
**6**	Lacasse et al. 2021	88y/M	Bacteraemia, upper limb erysipelas	Pure	CKD, CHD, skin malignancy, CLL	-	AMO	CAZ (10d)	Survived
**7**	Lee et al. 2021	62y/F	Bacteraemia, CRBSI, purulent breast wound	Pure	Bilateral invasive breast cancer	Purulent discharge from breast wound cultured *Corynebacterium striatum* and *Alcaligenes faecalis *.	CEP	TZP (14d), and removal of central line	Survived
**8**	Our case 2023	87y/F	Bacteraemia, empyema, purulent breast wound	Pure	Locally invasive breast cancer, CHD, DM, CKD	Purulent blood stained discharge from breast wound (culture not sent).	CRO/AZM (2d)	TZP (7d) -> withdrawal of active management	Death

– No data.

*BKA, below knee amputation; DM, diabetes mellitus; CKD, chronic kidney disease; CHD, coronary heart disease; CHF, congestive heart failure; HTN, hypertension; CRBSI, catheter related bloodstream infection.

†AMI, amikacin; AMC, amoxicillin-clavulanic acid; AMO, amoxicillin; AZM, azithromycin; CAZ, ceftazidime; CRO, ceftriaxone; CEP, cefepime; CIP, ciprofloxacin; CLI, clindamycin; CZ, cefazolin; CL, colistin; IMI, imipenem; MEM, meropenem; NET, netilmicin; TZP, piperacillin-tazobactam; SXT, sulfamethoxazole-trimethoprim; TOB, tobramycin; VAN, vancomycin.

‡Trans-M, transgender-male (identifies as female).

Host factors such as diabetes mellitus and underlying malignancy featured in most reported cases, reflecting a relatively susceptible host status as a pre-requisite for acquiring *B. trematum* infection. Wounds from long-standing ulcers or vascular exit sites have been hypothesized as likely entry sites into the bloodstream. Whilst colonization from the immediate environment has been proposed and transient host colonization is plausible, environmental niches have not been fully identified to truly establish the nature of this organism’s portal entry into a human host[18].

In our case, the recovery of *B. trematum* was unlikely an environmental contaminant or colonizing flora, and we believe it had contributed to our patient’s clinical outcome. Firstly, we recovered our isolates from two different sterile sites in pure culture, where each sample was collected independent from each other, 2 days apart. One was from the admission blood culture and the other from a day 3 pleural tap. Secondly, each sample was processed independent of each other in the laboratory, and at different time points. We believe the likely portal entry of *B. trematum* is the discharging left breast wound, given the contiguous anatomical site. However, no microbiological samples were obtained from this site nor any additional imaging performed to demonstrate direct invasion into the pleural space to support our suspicion. We recognize that *B. trematum* has true potential as an opportunistic invasive pathogen in an immunocompromised host, as evident in our case who was relatively immunocompromised secondary to advanced age, breast cancer, diabetes and chronic kidney disease.

MALDI-TOF and 16S RNA sequencing are methods for reliable identification of *B. trematum* in a clinical laboratory. Biochemical identification systems had led to misidentification in the past [2, 3, 5], which may have contributed to *B. trematum* isolates being overlooked in clinical specimens. With more clinical laboratories adopting the MALDI-TOF as their primary microbial identification system, *B. trematum* could be more readily and rapidly recognized. However, due to its rarity of occurrence from clinical sites and its relatively unknown pathogenicity, the reporting laboratory may dismiss it from mixed wound cultures, or may elect for confirmatory testing potentially delaying clinical reporting [19].

To date, there has not been clear consensus on specific antibiotic for treatment. Published susceptibility data for *B. trematum* are limited to case reports and studies to derive meaningful conclusion on treatment outcomes. In addition, the lack of species-specific breakpoints, different laboratory methodologies (i.e. disc diffusion, gradient strip diffusion, broth microdilution) and standards varied across published reports. Categorical susceptibilities reported in the literature were based on pharmacokinetic-pharmacodynamic (PK-PD) breakpoints from European Committee on Antimicrobial Susceptibility Testing (EUCAST), non-Enterobacterales CLSI breakpoints or Food and Drug Administration (FDA), to guide clinicians on therapeutic options. Whilst the mainstay of treatment for infected ulcers and wounds is surgical intervention, bloodstream and other sterile site infections will warrant systemic antibiotic treatment. Table 3 summarizes published bacteraemia cases and their phenotypic susceptibility profiles.

**Table 3.** Reported antibiotic susceptibilities of *B. trematum* from blood culture isolates

Antibiotic (MIC µg/ml, or MID mm)	Halim *et al*. 2014	Saksena *et al*. 2015	Majewski *et al.* 2016	Desurmont *et al*. 2018	Hummel *et al*. 2020	Lacasse *et al*. 2021	Lee *et al*. 2021	Our case 2023
**Methodology (breakpoints**)	DD (-)	Vitek-2, GSD (CLSI)	DD, BMD (CLSI, FDA)	GSD (EUCAST PK-PD)	–	–	Vitek-2 (CLSI)	BMD, GSD (CLSI)
Amoxicillin	–	–	–	S (1)	–	–	–	–
Ampicillin	–	–	–	–	–	–	–	NB (8)
Ampicillin-sulbactam	–	S (8)	–	–	–	–	–	NB (<=8)
Amoxicillin-clavulanic acid	R	–	S (23 mm)	S (1)	–	–	–	NB (4)
Piperacillin	–	I (64)	–	–	–	S	–	–
Piperacillin-tazobactam	–	S (8)	S (2)	S	–	S	S (<=4/4)	S (4)
Cefuroxime	–	–	R (no zone)	R	–	–	–	NB (>16)
Cefotaxime	R	–	I (16)	–	–	R	–	R (32)
Ceftriaxone	–	I (32)	–	R	–	–	–	–
Ceftazidime	R	R (64)	S (8)	–	–	S	–	S (8)
Cefepime	–	I (16)	–	–	–	S	R (64)	I (16)
Cefoxitin	–	–	–	R	–	–	–	R (>16)
Aztreonam	–	–	–	R	–	R	–	R (>16)
Ertapenem	–	–	–	–	–	–	–	NB (<=0.5)
Imipenem	S	S (1)	S (0.25)	–	–	S	–	S (<=2)
Meropenem	–	R (16)	–	–	–	S	–	S (<=1)
Ciprofloxacin	S	S (1)	S (0.008)	S (1)	–	–	–	I (2)
Levofloxacin	–	I (4)	S (0.03)	–	–	–	–	I (4)
Moxifloxacin	–	–	–	–	–	–	–	NB (>2)
Amikacin	R	R (64)	S (18 mm)	–	–	S	–	S (16)
Gentamicin	R	R (16)	R (11 mm)	–	–	I	–	I (8)
Netilmicin	S	–	–	–	–	R	–	–
Tobramycin	R	I (8)	S (15 mm)	–	–	S	–	I (8)
Erythromycin	–	–	–	NB (16)	–	–	–	–
Clarithromycin	S	–	–	–	–	–	–	–
Azithromycin	–	NB (2)	–	–	–	–	–	NB (4)
Trimethoprim-sulfamethoxazole	–	S (1/19)	–	R	–	–	–	S (<=2/38)
Tetracycline	S	–	–	NB (2)	–	–	–	S (<=4)
Colistin	S	R (16)	–	–	–	–	–	–

MIC, minimum inhibitory concentration; MID, minimum inhibitory diameter.

DD, disc diffusion; GSD, gradient strip diffusion (E-test); BMD, broth microdilution, Vitek-2 (Biomerieux).

CLSI, Clinical Laboratory Standards Institute; FDA, Food and Drug Administration, Institute; EUCAST, European Committee on Antimicrobial Susceptibility Testing; PK-PD, pharmacokinetic-pharmacodynamic.

– No data, *S* susceptible, *I* intermediate, *R* resistant, NB no breakpoint

Buechler *et al*. [20] recognized the scarcity of published susceptibility data, and presented the molecular and phenotypic susceptibility profile of three unique clinical *B. trematum* isolates. Our *B. trematum* isolates demonstrated *in vitro* susceptibility with piperacillin-tazobactam, meropenem and imipenem, but exhibited reduced susceptibility to the cephalosporins, aminoglycosides and fluoroquinolones tested, echoing the phenotypic susceptibility findings by Buechler *et al*. The team had also identified the fluoroquinolone and aminoglycoside resistance genes in its isolates, and a beta-lactamase encoding gene. In light of this, we had followed on with additional phenotypic testing to detect the presence of extended spectrum beta-lactamase (ESBL) by combined-disc testing (with cefotaxime and cefotaxime-clavulanate, and ceftazidime and ceftazidime-clavulanate), but did not detect the presence of ESBL in our isolate.

There may be limitations in the methodology used in susceptibility testing, as seen between our two *B. trematum* isolates. The one dilution difference in the cephalosporins and aminoglycosides, and the discordant results of the fluoroquinolones (levofloxacin and moxifloxacin) could be from intra-laboratory variations, or potentially, inherent challenges of susceptibility testing and interpretation with this organism.

Five of the eight bacteraemia cases reported had piperacillin-tazobactam as part of, or a definitive antibiotic treatment, of whom three survived. In our case, the *B. trematum* bloodstream infection was not sustained after a 7 day course of piperacillin-tazobactam, implies that other factors may have contributed to the patient’s clinical deterioration. Current *in vitro* data supports the use of carbapenems and to some extent piperacillin-tazobactam as empiric agents when faced with a clinical infection, but clinical outcome data is necessary before recommendations can be issued. However, in the absence of available susceptibilities, we would probably not recommend cephalosporins, aminoglycosides or fluoroquinolones as first-line agents.

## Conclusion

Invasive *B. trematum* infection remains uncommon but is associated with significant mortality in the immunocompromised. Consensus for antibiotic treatment remains unclear, with limited susceptibility data to support specific antibiotic use. We predict that this opportunistic pathogen will be reported more readily from clinical specimens with improved microbial identification systems, as well as enhanced clinical awareness. Standardized and more robust susceptibility works are needed to provide clear recommendations and establish consensus in treating invasive infections.
